# *HLA-G* Haplotypes Are Differentially Associated with Asthmatic Features

**DOI:** 10.3389/fimmu.2018.00278

**Published:** 2018-02-23

**Authors:** Camille Ribeyre, Federico Carlini, Céline René, François Jordier, Christophe Picard, Jacques Chiaroni, Laurent Abi-Rached, Philippe Gouret, Grégory Marin, Nicolas Molinari, Pascal Chanez, Julien Paganini, Delphine Gras, Julie Di Cristofaro

**Affiliations:** ^1^UMR7268 Anthropologie bio-culturelle, Droit, Ethique et Santé (ADES), “Biologie des Groupes Sanguins”, Aix Marseille Université, CNRS, Établissement Français du Sang (EFS), Marseille, France; ^2^Department of Immunology, CHRU de Montpellier, University Hospital Saint-Eloi, Montpellier, France; ^3^Faculté de Médecine, University of Montpellier 1, Montpellier, France; ^4^Établissement Français du Sang Alpes Méditerranée, Marseille, France; ^5^Equipe ATIP, URMITE UM63 CNRS 7278 IRD 198 INSERM 1095, IHU Méditerranée Infection, Aix Marseille Université, Marseille, France; ^6^Xegen, Gemenos, France; ^7^Institut Montpelliérain Alexander Grothendieck, CNRS, University of Montpellier, Montpellier, France; ^8^Department of Statistics, University of Montpellier Hospitals, Montpellier, France; ^9^Clinique des Bronches, Allergie et Sommeil, AP-HM Hôpital Nord, Marseille, France; ^10^INSERM U1067, CNRS UMR 7333, Aix Marseille Université, Marseille, France

**Keywords:** human leukocyte antigen-G, regulatory regions, haplotypes, asthma, phylogeny, alternative splicing, transcription factor binding site

## Abstract

Human leukocyte antigen (HLA)-G, a HLA class Ib molecule, interacts with receptors on lymphocytes such as T cells, B cells, and natural killer cells to influence immune responses. Unlike classical HLA molecules, HLA-G expression is not found on all somatic cells, but restricted to tissue sites, including human bronchial epithelium cells (HBEC). Individual variation in HLA-G expression is linked to its genetic polymorphism and has been associated with many pathological situations such as asthma, which is characterized by epithelium abnormalities and inflammatory cell activation. Studies reported both higher and equivalent soluble HLA-G (sHLA-G) expression in different cohorts of asthmatic patients. In particular, we recently described impaired local expression of HLA-G and abnormal profiles for alternatively spliced isoforms in HBEC from asthmatic patients. sHLA-G dosage is challenging because of its many levels of polymorphism (dimerization, association with β2-microglobulin, and alternative splicing), thus many clinical studies focused on *HLA-G* single-nucleotide polymorphisms as predictive biomarkers, but few analyzed *HLA-G* haplotypes. Here, we aimed to characterize *HLA-G* haplotypes and describe their association with asthmatic clinical features and sHLA-G peripheral expression and to describe variations in transcription factor (TF) binding sites and alternative splicing sites. *HLA*-*G* haplotypes were differentially distributed in 330 healthy and 580 asthmatic individuals. Furthermore, *HLA-G* haplotypes were associated with asthmatic clinical features showed. However, we did not confirm an association between sHLA-G and genetic, biological, or clinical parameters. *HLA-G* haplotypes were phylogenetically split into distinct groups, with each group displaying particular variations in TF binding or RNA splicing sites that could reflect differential HLA-G qualitative or quantitative expression, with tissue-dependent specificities. Our results, based on a multicenter cohort, thus support the pertinence of *HLA-G* haplotypes as predictive genetic markers for asthma.

## Introduction

Human leukocyte antigen-G (HLA-G), a member of the HLA class Ib (non-classical) family, modulates natural killer (NK) cells and cytotoxic T-lymphocyte-mediated activity as well as B-lymphocyte proliferation. HLA-G is also involved in epithelial cell differentiation (1–3).

Human leukocyte antigen-G is expressed by a restricted pattern of cells including human bronchial epithelium cells (HBEC) and displays interindividual variation expression in physiological conditions. The mechanisms responsible for tissue-specific expression and interindividual HLA-G expression variation are of utmost importance since HLA-G expression is associated with many diseases involving immune system surveillance and inflammatory disease, such as asthma (3, 4).

Asthma is characterized by structural changes in the airways with abnormalities of the epithelium and concomitant recruitment, survival, and activation of inflammatory cells. In asthma, soluble HLA-G (sHLA-G) expression was reported to be higher in bronchoalveolar lavage (BAL) from asthmatic individuals and in both plasma and serum from atopic asthmatic children, whereas others reported no difference in non-allergic asthmatics (5–8). In BAL from asthmatic patients, however, sHLA-G expression was reported to be inversely correlated with markers of asthma inflammation (9). Our group reported an impairment of full-length protein HLA-G expression in HBEC from asthmatic patients and abnormal expression of alternatively spliced membrane-bound (HLA-G1 to 4) and soluble (HLA-G5 to 7) forms (10). Both HLA-G1 and -G5 isoforms reduced NK cell cytotoxicity with an additive effect, and HLA-G2, -G4, and -G6 were described as functional, whereas conflicting results were published concerning HLA-G3 (11–14).

Human leukocyte antigen-G tissue-specific expression is reported to be driven by a 12 kb upstream enhancer site, defined as cis-regulatory Enhancer L (15). Concerning interindividual variation expression, HLA-G*01:04 has been associated with high HLA-G secretion and G*01:03 and G*01:05N with low HLA-G secretion (3). In the 3′ untranslated region (UTR), conflicting results were reported concerning association of HLA-G expression and the ex8 ins/del polymorphism (16); the 3142G allele was identified as a binding site for the microRNA-152 family (17) and 3187A/G and 3196C/G were reported to influence mRNA stability. 3′UTR haplotypes differentially influenced HLA-G expression in gene reporter assays conducted *in vitro* (18). However, *in silico* analysis of 3′UTR haplotypes suggested that most microRNAs targeting *HLA-G* in a strong or specific manner are not influenced by 3′UTR single-nucleotide polymorphisms (SNPs) and that 3′UTR haplotypes display equivalent means of microRNA binding (19). Polymorphism in the 5′ upstream regulatory region (URR) within regulatory elements, such as -1305G>A, -964G>A 725C>G>T, -716G>T and -56C>T, were reported to impact HLA-G expression (3, 20).

Human leukocyte antigen-G protein dosage has its drawbacks, and therefore, many teams have studied the association between *HLA-G* SNP and pathological situations (3). Few studies have focused on *HLA-G* genetic haplotypes as predictive biomarkers (21). *HLA-G* haplotypes are defined as SNPs being in Linkage Disequilibrium; Castelli et al. first described a restricted number of *HLA-G* haplotypes using SNPs in the 5′URR, 3′UTR, and coding regions in a Brazilian population (22). This low variability suggests a stabilizing selective effect on *HLA-G* haplotypes which could reflect a synergic effect of HLA-G SNPs. We previously characterized a deleterious association of two *HLA*-*G* haplotypes, HLA-G*01:04-UTR3 and HLA-G*01:06-UTR2, in lung transplantation outcome and in lung inflammatory diseases such as cystic fibrosis (23). Although the *HLA-G* gene was identified as an asthma susceptibility gene and SNPs -964 and 3142 were associated with asthma (17, 24–27), no study so far has described the haplotypes effect, shown to be more relevant than SNPs to predict sHLA-G dosage (28, 29), in asthma clinical studies.

We aimed to investigate the association of *HLA-G* haplotypes and sHLA-G peripheral expression with clinical features of asthma. We then aimed to analyze phylogenetic relationships, variations at potential transcription factor (TF) binding sites and alternative splicing sites between *HLA-G* haplotypes that could account for their differential association with asthmatic features.

## Materials and Methods

### Samples

This study was carried out in accordance with the French Public Health Code (art L1221-1). All subjects gave written informed consent in accordance with the Declaration of Helsinki. Three hundred and thirty volunteer bone marrow donors from France were recruited, blood donations were collected, and DNA was extracted from a 200 µl total blood sample using the QIAmp Blood DNA Mini kit (Qiagen, France) in accordance with BSL-2 practices. A medical interview was carried out prior to blood donation to exclude donors with medical contraindications such as severe asthma, respiratory failure, or allergies. Five hundred and ninety asthmatic patients enrolled in a French asthmatic multicenter cohort (*Cohorte Obstruction Bronchique et Asthme*, INSERM Hôpital Bichat, CNIL 28/01/2008) were included in the study. DNA samples with known genotype were used as control. Biological samples included 590 samples of DNA, 582 of serum, and a further 528 of serum collected 1 year after the first sample. Patient data were categorized into biological and clinical data and asthma severity according to the Guidelines for the Diagnosis and Management of Asthma, NHLBI 2007. Healthy individuals (HI) and asthmatic patient characteristics are detailed in Table 1.

**Table 1:** Healthy individuals (HI) and asthmatic patients’ [*Cohorte Obstruction Bronchique et Asthme* (COBRA)] characteristics (NA, non-applicable).

Patient data	HI	COBRA
Number of patients	330	590
Age (years) (median and range)	41 (18–59)	33 (16–83)
Sex (ratio)	1.60	1.75
Associated pathologies, ≥1 (%)	0	49
Hospitalization for asthma within the last 12 months, =0, =1, ≥2 (%)	0	78, 15, 7
Reanimation history for asthma (%)	0	21
Emergency consultation for asthma within the last 12 months, ≥1 (%)	0	45
Exacerbations within the last 12 months, ≥1 (%)	0	65
Blood eosinophils/mm^3^ (median and range)	NA	321 (0–3,321)
FEV2 (median and range)	NA	79 (18–463)
Allergy (%)	0	68
Control of asthma, controlled, partly controlled, and uncontrolled (%)	NA	26, 31, 43
Asthma control test score (median and range)	NA	14 (4–25)

### *HLA-G* Next Generation Sequencing

The *HLA-G* gene was sequenced from position −1983 to +3447 by next-generation sequencing (NGS). A 5,430 pb long amplicon was generated by PCR; primer sequences were 5′AGGAGCTGACACAGGAGGAA3′ and 5′CAGCTGAGCAGTGACCACAT3′; and amplification was performed using the Long Range PCR Kit (Qiagen, France). PCR fragments were sequenced using an NGS platform (MiSeq, Illumina, The Netherlands).

### *HLA-G* Allele and UTR Typing and Full-Length Sequence Regulatory Region Analysis

*HLA-G* NGS data were analyzed through PolyPheMe, specific software developed by the Xegen Company (France). HLA-G allelic assignment at eight digits was based on the HLA sequences listed in the official IMGT/HLA database (30). Regulatory regions were typed at low and high resolutions. Both low and high resolutions of regulatory region haplotypes were defined using PHASE and an EM algorithm implemented in the Gene[Rate] computer tools (31). Low-resolution typing (UTR 1–8) was performed as previously described (29) according to eight SNPs (-725/rs1233334, -716/rs2249863, -201/rs1233333, -56/rs17875397, ins/del exon 8/rs66554220, 3142/rs1063320, 3187/rs9380142, and 3196/rs1610696). High-resolution typing of regulatory regions (H1 to H74) was performed according to all polymorphic variations from position −1983 to −1 in 5′URR and from +2540 to +3447 in 3′UTR. *HLA-G* haplotype identification numbers were coded as previously described (21). Allelic, UTR, and haplotype frequencies were estimated using PHASE and an EM algorithm implemented in the Gene[Rate] computer tools (31).

### Serum sHLA-G Protein Measurement

Soluble HLA-G (sHLA-G), i.e., peripheral HLA-G expression, was quantified in sera from 582 asthmatic patients and 528 sera collected 1 year after the first sample. All serum samples were stored at −80°C prior to analysis. Measurement of soluble isoforms sHLA-G1 and sHLA-G5 was performed in duplicate on serum samples using the ELISA test (Biovendor^®^, Czech Republic; capture antibody: MEM-G/9) according to the manufacturer’s protocol.

### Association of *HLA-G* Alleles and Haplotypes with Asthmatic Features and Peripheral sHLA-G Dosage

Missing data led to the exclusion of the concerned sample from further analyses. No multiple imputation was used. Genetic data frequencies between HI and asthmatic patients were compared with Chi-square tests.

Associations between patients’ asthmatic features, sHLA-G expression, and genetic polymorphism were tested with logistic regressions when the predicted variable was categorical and general linear regression when the predicted variable was quantitative. Univariate analyses were performed on each of these asthmatic features: eosinophil count (/mm^3^), history of near-fatal asthma, asthma control, asthma control test score, asthmatic exacerbations within the last 12 months, emergency consultation for asthma within the last 12 months, hospitalization within the last 12 months because of asthma, intensive care unit stays, according to biological parameters [gender, body mass index (BMI), occupational exposure, tobacco use, allergies, maternal asthma, and paternal asthma], *HLA-G* predictors (*HLA-G* alleles, *HLA-G* regulatory regions typed at low resolution, and *HLA-G* regulatory regions typed at high resolution), and sHLA-G serology and differences between sHLA-G dosage results in sera collected at a 1-year interval. Univariate analyses were also performed on sHLA-G dosage and differences between sHLA-G dosage results in sera collected at a 1-year interval according to clinical and biological parameters such as gender, height, weight, BMI, occupational exposure, tobacco use, allergies, associated pathologies, maternal asthma, paternal asthma, treatment for asthma or for allergy (CO intake, anticholinergics, anti-IgE, anti-leukotrienes, theophylline, antihistamines, anti-RGO, antihypertensives, statins, and fibrates), month of serum collection, and genetic parameters (*HLA-G* alleles, *HLA-G* UTR, and *HLA-G* haplotypes). The False Discovery Rate correction for multiplicity was applied; however, no power analysis was performed. Variables with *p* values below 0.05 and corrected *p* values below 0.20 in univariate analysis were further investigated in multivariate analysis. Association results with quantitative variables are expressed as estimates and standard errors (Std error), whereas association results with qualitative variables are expressed as odd ratios (OR) and 95% confidence intervals.

### *HLA-G* Full-Length Sequence Phylogenetic Analysis

*HLA-G* full-length sequences from position −1983 to +3447 were aligned using the multiple sequence alignment tool MUSCLE in the Molecular Evolutionary Genetics Analysis (MEGA) software version 7.0 (32). Evolutionary analyses were conducted in MEGA7. Evolutionary relationships among *HLA-G* full-length sequences were inferred using the neighbor-joining (NJ) method with 1,000 replicates. Evolutionary distances were computed using the *p*-distance method, and units were the number of base differences per site. Analysis involved gorilla *HLA-G* genomic sequence as an outgroup (accession number CU104658.1).

### TF Binding Site Analysis and TF Expression in the Lungs and in Blood

Each SNP in the 5′UTR was analyzed for potential TF binding site variation with the AliBaba 2.1 and ConSite programs. TFs found to have differential binding sites between *HLA-G* haplotypes were checked for their tissue-specific expression (blood and lungs) in NCBI and Tissue-specific Gene Expression and Regulation (TiGER) databases (33). NCBI data included the “HPA RNA-seq normal tissues” project, i.e., RNA-seq performed on 27 different tissue samples from 95 human individuals and the “Illumina bodyMap2 transcriptome” project, i.e., transcription profiling by high-throughput sequencing of RNA from 16 different human tissues.

### Splicing Site Variation Analysis

Variation in splicing sites was studied for alleles sharing identical or similar UTR haplotypes using the Human Splicing Finder program (34). This program combines different algorithms to identify and predict the effect of mutations on acceptor and donor splice site motifs.

## Results

### *HLA-G* Haplotypes Are Differentially Distributed in Healthy and Asthmatic Individuals and Correlate with Asthmatic Features

Next generation sequencing typing was successful in 317 (96%) HI and 582 (98%) asthmatic patients. Thirty-seven SNPs, 1 base deletion, 1 base insertion, and a 14bp insertion were validated through Xegen pipeline analysis in the regulatory regions 5′URR and 3′UTR from position −1983 to +3447; all were previously described (30, 35, 36). *HLA-G* sequencing data have been submitted to the NCBI public data base (Table 2).

**Table 2:** *HLA-G* regulatory region haplotypes identifying number (ID), nucleotide variations, untranslated region (UTR) and allelic association, and frequencies estimated by EM algorithm in healthy individuals (HI) and asthmatic patients [*Cohorte Obstruction Bronchique et Asthme* (COBRA)] with a total frequency higher than 0.3% estimated by the EM algorithm: ID, nucleotide variations, UTR and allelic association, and frequencies in HI and asthmatic patients (COBRA).

Haplotype ID	Nucleotides variations, UTR and allele association																																									GenBank accession number	HI (%)	COBRA (%)	*p*-value
	-1898 T>C	-1746 C>A	-1573 T>C	-1305 G>A	-1179 A>G	-1155 G>A	-1140 A>T	-1138 A>G	-1121 C>T	-964 A>G	-762 T>C	-725 C>G>T	-716 G>T	-689 G>A	-666 T>G	-646 A>G	-633 A>G	-546 *>G	-540 A>*	-509 C>G	-486 C>A	-483 A>G	-477 G>C	-400 G>A	-391 G>A	-369 A>C	-201 A>G	-56 C>T	2798 G>A	2804 G>T	2838 C>Gex	8 D>I	3003 T>C	3010 C>G	3027 C>T	3035 C>T	3142 C>G	3187 A>G	3196 C>G	3227 G>A

H01	T	C	T	G	A	G	A	A	C	G	C	C	T	A	G	A	G	*	A	C	A	A	C	G	G	C	G	C	G	G	C	D	T	G	C	C	C	G	C	G	UTR1 G*01:01:01:01	MG825364	165 (26)	291 (25)	0.633
H05	T	C	T	G	A	G	A	A	C	G	C	C	T	A	G	A	G	*	A	C	A	G	C	G	G	C	G	C	G	G	C	D	T	G	C	C	C	G	C	G	UTR1 G*01:01:01:01	MG825353	11 (1.73)	33 (2.83)	0.149
H54	T	C	T	G	A	G	A	A	C	G	C	C	T	A	G	A	G	G	A	C	A	A	C	G	G	C	G	C	G	G	C	D	T	G	C	C	C	G	C	G	UTR1 G*01:01:01:01	MG825355	2 (0.31)	5 (0.42)	0.71
H10	C	A	C	A	G	G	T	A	C	A	T	C	G	G	T	A	A	*	A	C	C	A	G	G	G	A	A	C	A	T	G	I	T	C	C	C	G	A	G	G	UTR2 G*01:01:02:01	MG825359	125 (19.7)	232 (19.9)	0.912
H10	C	A	C	A	G	G	T	A	C	A	T	C	G	G	T	A	A	*	A	C	C	A	G	G	G	A	A	C	A	T	G	I	T	C	C	C	G	A	G	G	UTR2 G*01:06	MG825360	36 (5.67)	87 (7.47)	0.149
H10	C	A	C	A	G	G	T	A	C	A	T	C	G	G	T	A	A	*	A	C	C	A	G	G	G	A	A	C	A	T	G	I	T	C	C	C	G	A	G	G	UTR2 G*01:05N	MG825361	16 (2.52)	25 (2.14)	0.609
H10	C	A	C	A	G	G	T	A	C	A	T	C	G	G	T	A	A	*	A	C	C	A	G	G	G	A	A	C	A	T	G	I	T	C	C	C	G	A	G	G	UTR2 G*01:01:02:02	MG825362	3 (0.47)	8 (0.68)	0.578
H23	T	C	C	A	G	A	A	A	C	A	T	C	G	G	T	A	A	*	A	C	C	A	G	G	G	A	A	C	G	T	G	D	T	C	C	C	G	A	C	G	UTR3 G*01:04:01	MG825349	41 (6.46)	50 (4.29)	**0.044**
H23	T	C	C	A	G	A	A	A	C	A	T	C	G	G	T	A	A	*	A	C	C	A	G	G	G	A	A	C	G	T	G	D	T	C	C	C	G	A	C	G	UTR3 G*01:04:04	MG825348	9 (1.41)	40 (3.43)	**0.012**
H04	T	C	T	G	A	G	A	A	T	G	C	G	T	A	G	A	G	*	A	C	A	A	C	G	G	C	G	C	G	G	C	D	C	G	C	C	C	A	C	G	UTR4 G*01:01:01:05	MG825356	51 (8.04)	60 (5.15)	**0.015**
H02	T	C	T	G	A	G	A	A	C	G	C	G	T	A	G	A	G	*	A	C	A	A	C	G	G	C	G	C	G	G	C	D	C	G	C	C	C	A	C	G	UTR4 G*01:01:01:05	MG825358	44 (6.94)	48 (4.12)	**0.009**
H21	T	C	C	G	G	G	A	G	C	G	C	T	T	A	G	G	G	*	A	G	A	A	G	A	A	A	G	T	G	T	G	I	T	C	C	T	G	A	C	G	UTR5 G*01:03:01:02	MG825350	10 (1.57)	31 (2.66)	0.14
H19	T	C	C	G	G	G	A	G	C	G	C	T	T	A	G	A	G	G	A	C	A	A	G	A	A	A	G	T	G	T	G	I	T	C	C	T	G	A	C	G	UTR5 G*01:03:01:02	MG825357	9 (1.41)	14 (1.2)	0.695
H20	T	C	C	G	G	G	A	G	C	G	C	T	T	A	G	A	G	G	A	G	A	A	G	A	A	A	G	T	G	T	G	I	T	C	C	T	G	A	C	G	UTR5 G*01:03:01:02	MG825354	11 (1.73)	9 (0.77)	0.063
H03	T	C	T	G	A	G	A	A	C	G	C	C	T	A	G	A	G	*	*	C	A	A	C	G	G	C	G	C	G	G	C	D	T	G	C	C	C	A	C	A	UTR6 G*01:01:01:04	MG825363	25 (3.94)	62 (5.32)	0.191
H47	T	C	T	G	A	G	A	A	C	G	C	C	T	A	G	A	G	*	*	C	A	A	C	G	G	C	G	C	G	G	C	D	T	G	C	C	C	A	C	G	UTR6 G*01:01:01:04	MG825352	(0)	17 (1.46)	**0.002**
H46	T	C	T	G	A	G	A	A	C	G	C	C	T	A	G	A	G	*	A	C	A	A	C	G	G	C	G	C	G	G	C	D	T	G	C	C	C	A	C	G	UTR6 G*01:01:01:01	MG825351	3 (0.47)	9 (0.77)	0.455
H49	T	C	T	G	A	G	A	A	C	G	C	C	T	A	G	A	G	*	A	C	A	A	C	G	G	C	G	C	G	G	C	D	C	G	C	C	C	A	C	G	UTR6 G*01:01:01:06	MG825365	1 (0.15)	10 (0.85)	0.068
H16	C	A	C	A	G	G	T	A	C	A	T	C	G	G	T	A	A	*	A	C	C	A	G	G	G	A	A	C	G	T	G	I	T	C	A	T	G	A	C	G	UTR7 G*01:01:03:03	MG825347	30 (4.73)	73 (6.27)	0.179

Total of haplotypes with frequency above 0.3%																																											592 (93.3)	1,104 (94.8)	

*Significant *p*-values (chi-square test) are in bold*.

*HLA-G* alleles were typed at maximum resolution (eight digits) and regulatory regions (UTR1–8) were typed at low resolution according to eight SNPs in 5′URR and at high resolution based on all 28 polymorphisms in the 5′URR and 12 polymorphisms in the 3′UTR. Fifteen haplotypes with frequency above 0.3% displayed 94% of the cumulated frequencies. Their description and frequencies are detailed in Table 2.

Whereas high-resolution typing of regulatory region haplotypes did not discriminate between low-resolution typed haplotypes (such as UTR2/H10, UTR3/H23, and UTR7/H16), some UTRs were refined (such as UTR1, UTR4, UTR5, and UTR6, respectively, split into three, two, two, and four haplotypes). Several eight-digit alleles were associated with one haplotype, except for G*01:01:01:01 and G*01:03:01:02, which were associated with several haplotypes, and G*01:01:03:03 and *UTR7/H16* that were exclusively associated together.

H23-UTR3-G*01:04:01 and H23-UTR3-G*01:04:04 were, respectively, higher and lower in HI than in asthmatic patients (*p* = 0.044 and *p* = 0.012); H04-UTR4-G*01:01:01:05 and H02-UTR4-G*01:01:01:05 were both higher in HI than in asthmatic patients (*p* = 0.015 and *p* = 0.009), finally H47-UTR6-G*01:01:01:04 was not observed in HI (*p* = 0.002) (Table 2).

Correlations between HLA-G alleles, UTR, or haplotypes and asthmatic patient data investigated in multivariate analysis are shown in Tables 3A,B. Variables included in the multivariate models were gender and UTR2 for asthmatic exacerbation; gender, age, tobacco use, and UTR7 for acute asthma recurrence; and BMI, age, maternal asthma, tobacco use, UTR2, and UTR3 for eosinophil count. UTR2 (*N* = 369) was associated with asthmatic exacerbation within the last 12 months [*p* = 0.011; OR = 1.62 (1.115–2.355)] and eosinophil count (/mm^3^) (*p* = 0.011; estimate = 73.275, Std error = 28.75). UTR7 (*N* = 76) was associated with acute asthma recurrence [*p* = 0.043; OR = 1.838 (1.019–3.313)].

**Table 3:** (A,B) Genetic variables with *p*-value < 0.05 in multivariate analysis associated with *Cohorte Obstruction Bronchique et Asthme* asthmatic patient data (OR, odds ratio; 95% CI, confidence interval 95%; and Std error, standard error).

(A)					

Asthmatic patient data	Variable	OR	95% CI		*p*-Value
Asthmatic exacerbation within the last 12 months	UTR2 1 vs. 0	1.62	1.115	2.355	0.011
History of near-fatal asthma	UTR7 1 vs. 0	1.838	1.019	3.313	0.043
**(B)**					

**Asthmatic patient data**	**Variable**	**Estimate**	**Std error**		***p*-Value**

Eosinophil count (/mm^3^)	UTR2 1	73.275	28.75		0.011

*Analyses were performed with logistic regressions when the predicted variable was categorical and general linear regression when the predicted variable was quantitative*.

### Peripheral sHLA-G Expression Is Not Associated with Genetics or Asthmatic Features

Serum sHLA-G measurements from asthmatic patients were 17.1 (0–493) UI/ml and 17.5 (0–404) UI/ml 1 year later (mean and range). Multivariate analyses revealed no association between peripheral sHLA-G expression and patients’ asthmatic features, biological and clinical data, or HLA-G genetics (data not shown).

### *HLA-G* Haplotype Variation Defines Four Main Groups of Sequences

**Figure 1:**
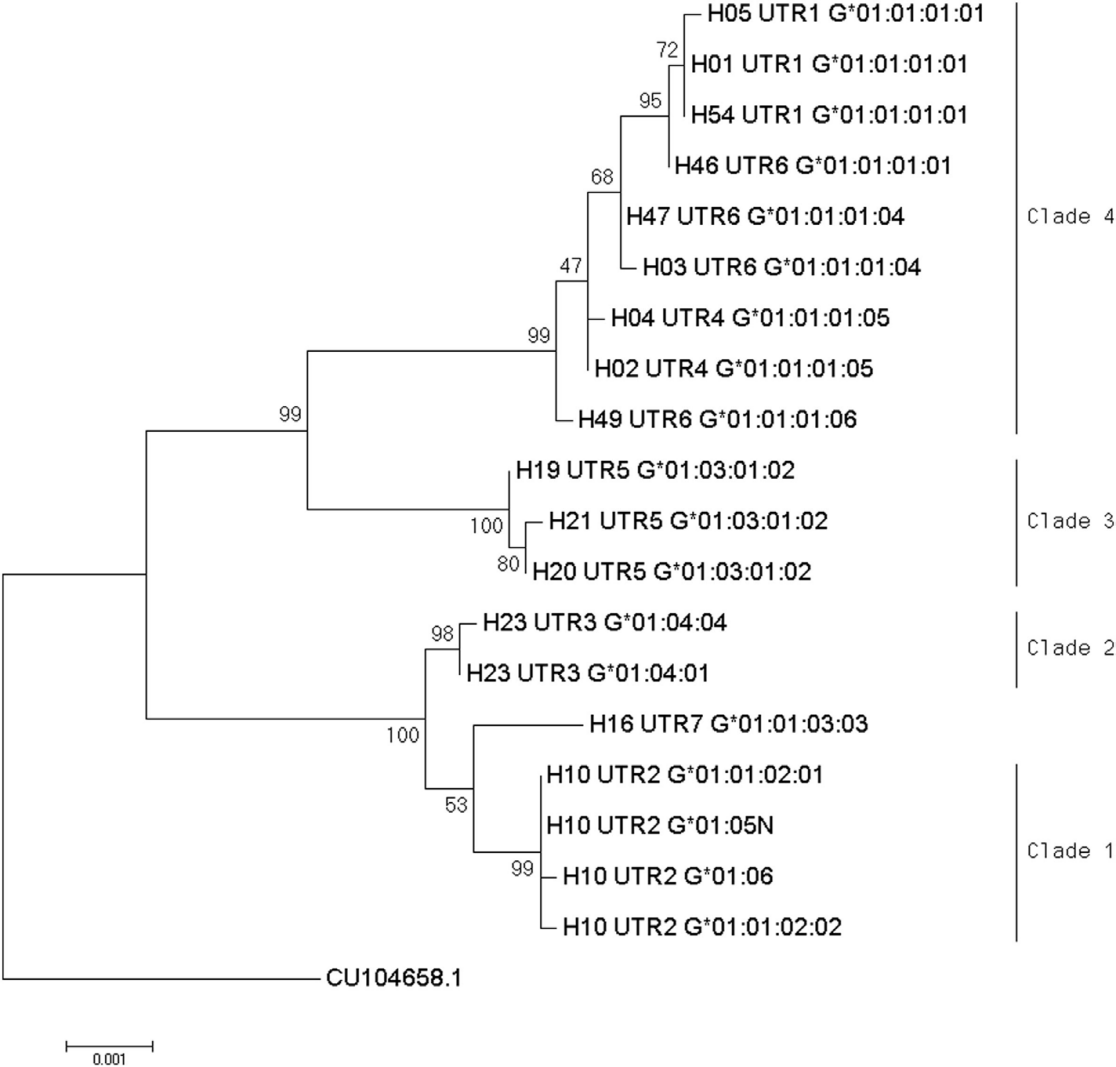
Phylogenetic relationship between human leukocyte antigen-G (HLA-G) allele-haplotype sequences. The phylogenetic tree contains 19 sequences from −1983 to +3447 aligned using the multiple sequence alignment tool MUSCLE in the Molecular Evolutionary Genetics Analysis software version 7.0; sequences were H01-UTR1-G*01:01:01:01, H05-UTR1-G*01:01:01:01, H54-UTR1-G*01:01:01:01, H04-UTR4-G*01:01:01:05, H02-UTR4-G*01:01:01:05, H21-UTR5-G*01:03:01:02, H20-UTR5-G*01:03:01:02, H19-UTR5-G*01:03:01:02, H10-UTR2-G*01:01:02:01, H10-UTR2-G*01:01:02:02, H10-UTR2-G*01:06, H10-UTR2-G*01:05N, H23-UTR3-G*01:04:01, H23-UTR3-G*01:04:04, H16-UTR7G*01:01:03:03, H46-UTR6-G*01:01:01:01, H47-UTR6-G*01:01:01:04, H03-UTR6-G*01:01:01:04, and H49-UTR6-G*01:01:01:06. Evolutionary relationships among HLA-G whole sequences were inferred using the neighbor-joining method with 1,000 replicates. Evolutionary distances were computed using the *p*-distance method, and units were the number of base differences per site. The percentage of trees in which the associated taxa clustered together is shown next to the branches. The sum of branch length equals 0.01616800 base differences per site. The percentage of replicate trees in which the associated taxa clustered together in the bootstrap test is shown next to the branches. The tree was drawn to scale, with branch lengths in the same units (base differences per site) as the evolutionary distances used to construct the phylogenetic tree. Analysis involved gorilla HLA-G genomic sequence as an outgroup (accession number CU104658.1).

Phylogenetic relationships for the 19 combinations of *HLA-G* alleles and haplotypes with a frequency above 0.3% were investigated using the NJ method (Figure 1). The resulting phylogenetic tree shows a clustering that follows UTR variation. Indeed, four main clades can be defined: the first clade contains the H10-UTR2 sequences (G*01:01:02:01, G*01:01:02:02, G*01:05N, and G*01:06), the second clade consists of the H23-UTR3 sequences (G*01:04:01 and G*01:04:04), the third clade includes UTR5-G*01:03:01:02 sequences (haplotypes H21, H20, and H19), and the fourth clade assembles the UTR6, UTR4, and UTR1 sequences. This fourth clade had the most divergent sequences (associated with G*01:01:01:01 and G*01:01:01:05 alleles) as compared to a reference gorilla HLA-G sequence (Table 4). H16-UTR7-G*01:01:03:03 sequence formed an isolated branch situated above clade 1.

**Table 4:** Pairwise distance between each haplotype calculated by Molecular Evolutionary Genetics Analysis program.

	CU104658.1	H16_UTR7_G*01:01:03:03	H23_UTR3_G*01:04:04	H23_UTR3_G*01:04:01	H21_UTR5_G*01:03:01:02	H46_UTR6_G*01:01:01:01	H47_UTR6_G*01:01:01:04	H05_UTR1_G*01:01:01:01	H20_UTR5_G*01:03:01:02	H54_UTR1_G*01:01:01:01	H04_UTR4_G*01:01:01:05	H19_UTR5_G*01:03:01:02	H02_UTR4_G*01:01:01:05	H10_UTR2_G*01:01:02:01	H10_UTR2_G*01:06	H10_UTR2_G*01:05N	H10_UTR2_G*01:01:02:02	H03_UTR6_G*01:01:01:04	H01_UTR1_G*01:01:01:01	H49_UTR6_G*01:01:01:06
CU104658.1		0.0014	0.0015	0.0015	0.0016	0.0015	0.0015	0.0015	0.0016	0.0015	0.0015	0.0016	0.0015	0.0015	0.0015	0.0015	0.0014	0.0015	0.0015	0.0015
H16_UTR7_G*01:01:03:03	0.0094		0.0007	0.0007	0.0015	0.0013	0.0013	0.0013	0.0015	0.0013	0.0013	0.0014	0.0013	0.0006	0.0006	0.0006	0.0006	0.0013	0.0013	0.0012
H23_UTR3_G*01:04:04	0.0093	0.0024		0.0002	0.0014	0.0012	0.0012	0.0012	0.0014	0.0012	0.0012	0.0014	0.0012	0.0005	0.0006	0.0005	0.0005	0.0012	0.0012	0.0011
H23_UTR3_G*01:04:01	0.0091	0.0022	0.0002		0.0014	0.0012	0.0012	0.0012	0.0014	0.0012	0.0012	0.0013	0.0012	0.0005	0.0005	0.0005	0.0005	0.0012	0.0012	0.0011
H21_UTR5_G*01:03:01:02	0.0098	0.0087	0.0085	0.0084		0.0013	0.0012	0.0013	0.0002	0.0013	0.0012	0.0002	0.0012	0.0013	0.0014	0.0014	0.0013	0.0012	0.0013	0.0012
H46_UTR6_G*01:01:01:01	0.0104	0.0091	0.0085	0.0084	0.0063		0.0003	0.0003	0.0012	0.0002	0.0005	0.0012	0.0004	0.0012	0.0013	0.0012	0.0012	0.0004	0.0002	0.0005
H47_UTR6_G*01:01:01:04	0.0100	0.0089	0.0084	0.0082	0.0058	0.0006		0.0004	0.0012	0.0004	0.0004	0.0012	0.0003	0.0012	0.0013	0.0012	0.0012	0.0002	0.0004	0.0004
H05_UTR1_G*01:01:01:01	0.0108	0.0095	0.0089	0.0087	0.0067	0.0004	0.0009		0.0013	0.0002	0.0005	0.0012	0.0005	0.0013	0.0013	0.0013	0.0013	0.0004	0.0002	0.0006
H20_UTR5_G*01:03:01:02	0.0098	0.0085	0.0084	0.0082	0.0002	0.0061	0.0056	0.0065		0.0013	0.0012	0.0002	0.0012	0.0013	0.0014	0.0013	0.0013	0.0012	0.0013	0.0011
H54_UTR1_G*01:01:01:01	0.0108	0.0093	0.0087	0.0085	0.0065	0.0002	0.0007	0.0002	0.0063		0.0005	0.0012	0.0004	0.0012	0.0013	0.0012	0.0012	0.0004	0.0000	0.0006
H04_UTR4_G*01:01:01:05	0.0108	0.0097	0.0091	0.0089	0.0063	0.0013	0.0007	0.0017	0.0061	0.0015		0.0012	0.0002	0.0013	0.0013	0.0013	0.0012	0.0004	0.0005	0.0005
H19_UTR5_G*01:03:01:02	0.0096	0.0083	0.0082	0.0080	0.0004	0.0059	0.0054	0.0063	0.0002	0.0061	0.0059		0.0011	0.0013	0.0014	0.0013	0.0013	0.0012	0.0012	0.0011
H02_UTR4_G*01:01:01:05	0.0106	0.0095	0.0089	0.0087	0.0061	0.0011	0.0006	0.0015	0.0059	0.0013	0.0002	0.0058		0.0013	0.0013	0.0013	0.0013	0.0004	0.0004	0.0004
H10_UTR2_G*01:01:02:01	0.0100	0.0020	0.0019	0.0017	0.0093	0.0093	0.0091	0.0097	0.0091	0.0095	0.0098	0.0089	0.0097		0.0002	0.0000	0.0002	0.0012	0.0012	0.0011
H10_UTR2_G*01:06	0.0102	0.0022	0.0020	0.0019	0.0094	0.0095	0.0093	0.0098	0.0093	0.0097	0.0100	0.0091	0.0098	0.0002		0.0002	0.0002	0.0013	0.0013	0.0012
H10_UTR2_G*01:05N	0.0100	0.0020	0.0019	0.0017	0.0093	0.0093	0.0091	0.0097	0.0091	0.0095	0.0098	0.0089	0.0097	0.0000	0.0002		0.0002	0.0012	0.0012	0.0011
H10_UTR2_G*01:01:02:02	0.0102	0.0022	0.0020	0.0019	0.0094	0.0095	0.0093	0.0098	0.0093	0.0097	0.0100	0.0091	0.0098	0.0002	0.0004	0.0002		0.0012	0.0012	0.0011
H03_UTR6_G*01:01:01:04	0.0102	0.0091	0.0085	0.0084	0.0059	0.0007	0.0002	0.0011	0.0058	0.0009	0.0009	0.0056	0.0007	0.0093	0.0095	0.0093	0.0095		0.0004	0.0005
H01_UTR1_G*01:01:01:01	0.0106	0.0093	0.0087	0.0085	0.0065	0.0002	0.0007	0.0002	0.0063	0.0000	0.0015	0.0061	0.0013	0.0095	0.0097	0.0095	0.0097	0.0009		0.0006
H49_UTR6_G*01:01:01:06	0.0104	0.0087	0.0082	0.0080	0.0059	0.0015	0.0009	0.0019	0.0058	0.0017	0.0009	0.0056	0.0007	0.0089	0.0091	0.0089	0.0091	0.0011	0.0017	

*Color shades depict higher (red) and lower (green) values. Standard errors are shown in blue*.

### TF Binding Site Variations between *HLA-G* Haplotypes

Thirty-four TFs were identified to bind differentially to each 5′URR sequence by *in silico* analysis (Tables 5 and 6), their classification is provided in Table 7; their specific expression in blood and lungs according to NCBI and TiGER databases is given in Table 6.

**Table 5:** 5′Upstream regulatory region polymorphism involved in the creation or the modification of factor transcription binding sites.

Position	Allele 1	Allele 2
-1898 T>C	None	TEF, SRF, IRF1
-1746 C>A	TBP, NF-E	FOX1, Lmo complex, GATA1
-1305 G>A	RUNX1	C/EBP
-1179 A>G	None	SP1
-1155 G>A	SOX5/PR	none
-1140 A>T	None	POU2F1
-1121 C>T	SP1	None
-964 A>G	NF-1, POU2F1, RXR, VDR, SP1	None
-725 C>G -716 G>T	None	C/EBP
-725 C>T -716 G>T	None	C/EBP, NF-1
-689 G>A	E2F, MYB	2 sites MYB
-666 T>G	XFD-1, NR2C1, SRF, POU2F1	None
-646 A>G	REL, PAX-4	Augmentation REL
-633 A>G	CDX1	RFX1
-546 *>G -540 A>*	POU2F1, SP1, PAX-4, IRF1, HNF1, MEF-2, C/EBP, IKZF1	POU2F1, SP1, PAX-4, IRF1, HNF1, MEF-2, C/EBP
-486 C>A	NR3C1, SP1, HNF4	None
-483 A>G	SP1, HNF4	None
-477 G>C	SP1, SREBF1	Diminution SREBF1
-400 G>A	2 sites SP1	1 site SP1
-391 G>A	STAT, 1 site ELK	TEF, 2 sites ELK
-369 A>C	SP1	2 sites SP1
-201 A>G	CUX1	None
-56 C>T	SP1	IRF1

**Table 6:** Transcription factor (TF) binding site variation between human leukocyte antigen-G (HLA-G) haplotypes (“0” describes a loss of binding site; “1” a gain of binding site the sites variation; “−”a binding site diminution; and “+” a binding site augmentation).

TF	Expression NCBI	Expression Tissue-specific Gene Expression and Regulation (TiGER)	H23 UTR3	H10 UTR2	H16 UTR7	H21 UTR5	H19 UTR5	H20 UTR5	H04 UTR4	H02 UTR4	H49 UTR6	H46 UTR6	H05 UTR1	H01 UTR1	H54 UTR1	H03 UTR6	H47 UTR6
SREBF1	wBc/L	B/L	1	1	1	1	1	1	1(−)	1(−)	1(−)	1(−)	1(−)	1(−)	1(−)	1(−)	1(−)
RFX1	wBc/L	L	0	0	0	1	1	1	1	1	1	1	1	1	1	1	1
NFE2	wBc/L	B*/L	1	0	0	1	1	1	1	1	1	1	1	1	1	1	1
C/EBP	wBc/L	B/L	2	2	2	2	2	2	2	2	1	1	1	1	1	1	1
NF1	wBc/L	B/L	1	1	1	1	1	1	0	0	0	0	0	0	0	0	0
REL	wBc/L	B*/L	1	1	1	1(+)	1	1	1	1	1	1	1	1	1	2	2
MEF-2	wBc/L	L	1	1	1	1	1	1	1	1	1	1	1	1	1	0	0
SRF	wBc/L	B/L	1	2	2	0	0	0	0	0	0	0	0	0	0	0	0
RUNX1	wBc/L	B*/L	0	0	0	1	1	1	1	1	1	1	1	1	1	1	1
SOX5	L	B/L*	0	1	1	1	1	1	1	1	1	1	1	1	1	1	1
STAT	wBc/L	B/L	1	1	1	0	0	0	1	1	1	1	1	1	1	1	1
TBP	wBc/L	B/L	1	0	0	1	1	1	1	1	1	1	1	1	1	1	1
CDX1	x	x	1	1	1	0	0	0	0	0	0	0	0	0	0	0	0
CUX1	wBc/L	B/L	1	1	1	0	0	0	0	0	0	0	0	0	0	0	0
HNF1	wBc	na	1	1	1	1	1	1	1	1	1	1	1	1	1	0	0
Lmo complex	x	x	0	1	1	0	0	0	0	0	0	0	0	0	0	0	0
POU2F1	wBc/L	B/L	3	4	4	1	1	1	1	1	1	1	1	1	1	1	1
IRF1	wBc/L	B/L	1	1	1	2	2	2	1	1	1	1	1	1	1	1	1
MYB	wBc/L	B/L	1	1	1	2	2	2	2	2	2	2	2	2	2	2	2
ELK	wBc/L	B/L	1	1	1	2	2	2	1	1	1	1	1	1	1	1	1
FOXI1	x	B/L	0	1	1	0	0	0	0	0	0	0	0	0	0	0	0
XFD-1	wBc/L	B/L	1	1	1	0	0	0	0	0	0	0	0	0	0	0	0
E2F	wBc/L	L	1	1	1	0	0	0	0	0	0	0	0	0	0	0	0
TEF	wBc/L	L	0	1	1	1	1	1	0	0	0	0	0	0	0	0	0
PAX-4	x	na	2	2	2	1	2	2	2	2	2	2	2	2	2	1	1
GATA1	wBc/L	na	0	1	1	0	0	0	0	0	0	0	0	0	0	0	0
IKZF1	wBc/L	B*/L	1	1	1	1	0	0	1	1	1	1	1	1	0	1	1
SP1	wBc/L	B/L	10	10	10	7	7	7	7	8	6	8	7	8	8	8	8
GR	wBc/L	B/L	1	1	1	0	0	0	0	0	0	0	0	0	0	0	0
PR	L	B/L	0	1	1	1	1	1	1	1	1	1	1	1	1	1	1
HNF4	x	x	2	2	2	1	1	1	1	1	1	1	0	1	1	1	1
RXR	wBc/L	B*	1	1	1	0	0	0	0	0	0	0	0	0	0	0	0
NR2C1	wBc/L	x	1	1	1	0	0	0	0	0	0	0	0	0	0	0	0
VDR	wBc/L	B/L	1	1	1	0	0	0	0	0	0	0	0	0	0	0	0

TF expression in the lungs (L) or in blood (B) are shown according to NCBI and TiGER databases (L, expressed in the lungs; wBc, expressed in white blood cells; B, expressed in blood; x, no expression; na, not available; *p-value < 0.05).

**Table 7:** Transcription factors (TFs) identified to bind differentially to each 5′upstream regulatory region sequence, classification according to super class, class, and families.

TF	Superclass	Class	Family
*SREBP*	Basic domains	Helix-loop-helix/leucine Zipper factors (bHLHZIP)	Ubiquitous bHLH-ZIP factors
*RFX1*		Leucine zipper factors (bZIP)	AP-1(-like) components
*NF-E*		Leucine zipper factors (bZIP)	AP-1(-like) components
*C/EBP*		Leucine zipper factors (bZIP)	C/EBP-like factors
*NF-1*		NF-1	NF-1

*REL*	Beta-scaffold factors with minor groove contacts	RHR (Rel homology region)	Rel/ankyrin
*MEF-2*		MADS box	Regulators of differentiation
*SRF (AGL3)*		MADS box	Responders to external signals
*RUNX1 (AML1)*		Runt	Runt
*SOX5*		HMG	SOX
*STAT*		STAT	STAT
*TATA binding protein (TBP)*		TATA-binding proteins	TBP

*CDX1*	Helix-turn-helix	Homeodomain	Homeodomain only
*CUX1 (CDP-CR1)*		Homeodomain	Homeodomain only
*HNF1*		Homeodomain	Homeodomain only
*Lmo complex*		Homeodomain	Homeodomain with LIM region
*POU2F1 (Oct1)*		Homeodomain	POU domain factors
*IRF1*		Tryptophan clusters	Interferon-regulating factors
*MYB*		Tryptophan clusters	Myb
*ELK*		Tryptophan clusters	Ets-type
*FOXI1 (HFH-3)*		Fork head/winged helix	Other regulators
*XFD-1*		Fork head/winged helix	Developmental regulators
*E2F*		Fork head/winged helix	Cell-cycle controlling factors
*TEF*		TEA domain	TEA
*PAX-4*		Paired box	

*GATA1*	Zinc-coordinating DNA-binding domains	diverse Cys4 zinc fingers	GATA-Factors
*IKZF1 (Ikaros-1)*		Cys2His2 zinc finger domain	Developmental/cell cycle regulators
*SP1*		Cys2His2 zinc finger domain	Ubiquitous factors
*GR (NR3C1)*		Cys4 zinc finger of nuclear receptor type	Steroid hormone receptors
*PR (NR3C3)*		Cys4 zinc finger of nuclear receptor type	Steroid hormone receptors
*HNF4*		Cys4 zinc finger of nuclear receptor type	Thyroid hormone receptor-like factors
*RXR*		Cys4 zinc finger of nuclear receptor type	Thyroid hormone receptor-like factors
*TR2 (NR2C1)*		Cys4 zinc finger of nuclear receptor type	Thyroid hormone receptor-like factors
*VDR*		Cys4 zinc finger of nuclear receptor type	Thyroid hormone receptor-like factors

Interestingly, haplotype grouping based on their TF binding site profiles mirrored that observed on the phylogenetic tree (Table 6; Figure 1). Hence, haplotypes displayed different TF binding site profiles, notably in haplotype H23 (UTR3-G*01:04:01/G*01:04:04): the transition G>A at position −1155 impaired progesterone receptor (PR) and SOX5 binding sites. Besides this unique feature, this haplotype displayed similarities with H16 (UTR7-G*01:01:03:03) and H10 (UTR2-G*01:06/G*01:05N/G*01:01:02:XX). Comparing the most common sequences, some SNPs created new binding sites: one to two sites for SRF, one site for CUX1, two to three additional sites for POU2F1, one site for E2F, one site for glucocorticoid receptor, additional sites for SP1 (up to 10), one site for XFD-1, RXR, NR2C1, VDR, CDX1, and an additional site for HNF4. Conversely, some SNPs in H23, H16, and H10 disrupted binding sites for RUNX1, RFX1, and MYB. H16 and H10 differed from H23 and other haplotypes as their SNPs created a fixation site for Lmo complex, FOXI1, and GATA1 and disrupted a binding site for NF-E and TATA binding proteins.

Haplotypes H21, H19, and H20 (associated with UTR5-G*01:03:01:02) distinguished themselves by an additional fixation site for ELK and IRF1, and the disruption of a fixation site for STAT; H19 and H20 displayed a disruption of a IKZF1 fixation site. H21 displayed an increase in REL binding sites. Genetic polymorphism in H21, H19, and H20 created a site for NF-1 and SREBF1 which were also present in haplotypes H23, H16, and H10. H21, H19, H20, H16, and H10 had a genetic polymorphism that created a site for TEF. Haplotypes H21, H19, H20, H23, H16, H10, H04, and H02 displayed a fixation site for C/EBP.

Concerning haplotypes associated with UTR6 (G*01:01:01:01, G*01:01:01:04, G*01:01:01:06), H47 and H03 distinguished themselves by an additional site for REL and disruption of a fixation site for MEF-2. H46 and H49, H01, H05, and H54 and H04 and H02 displayed quite similar patterns, with the exception of H54 for which a fixation site for IKZF1 was disrupted, as for H19 and H20.

### Splicing Site Variation Analysis

Variation in splicing sites was investigated for alleles with identical coding sequences but with variations at a six- or eight-digit level and sharing identical or similar haplotypes.

HLA-G*01:04:01 and HLA-G*01:04:04 alleles differ at position 1827G>A in the last positions of exon 4; 1827A in HLA-G*01:04:04 created an exonic cryptic acceptor site (Table 8A).

**Table 8:** (A,B) Splicing site variation for G*01:01:04:01 and G*01:01:04:04 (A) or G*01:01:01:01, G*01:01:01:04, G*01:01:01:05, and G*01:01:01:06 (B).

(A)		

Allele and exonic cryptic sites	1827A, Acceptor aggggctgccggAG	
G*01:01:04:011 *827G*	Disrupted aggggctgccagAG	
G*01:01:04:04 *1827A*	Present	
**(B)**		

**Allele and intronic cryptic sites**	**99A, Acceptor ggagggaaacagCC**	**494A, Acceptor cggcccagggagCA**

G*01:01:01:01 *99G, 494A*	Disrupted ggagggaaacggCC	Present
G*01:01:01:04 *99G, 494A*	Disrupted ggagggaaacggCC	Present
G*01:01:01:05 *99A, 494A*	Present	Present
G*01:01:01:06 *99A, 494C*	Present	Disrupted cggcccagggcgCA

HLA-G*01:01:02:01 and HLA-G*01:01:02:02 alleles differ only at position 1016C>T in intron 3, this SNP did not have any impact on splicing site prediction.

HLA-G*01:01:01:01, HLA-G*01:01:01:04, HLA-G*01:01:01:05, and HLA-G*01:01:01:06 displayed eight variations in introns 2, 3 and 4 (99G>A, 126b G>A, 130T>C, 482T>C, 494A>C, 505*>C, 531G>C, 1147C>T). HLA-G*01:01:01:01, HLA-G*01:01:01:04, and HLA-G*01:01:01:06 had a disrupted intronic cryptic acceptor site as compared to HLA-G*01:01:01:05 due to 99G>A at the beginning of intron 2 and 494A>C at the beginning of intron 3 (Table 8B).

## Discussion

*HLA-G* genetic polymorphism is extensively described in different human populations worldwide. However, there are only limited data describing the synergic effect of these SNPs, i.e., haplotypes, in clinical studies.

We fully characterized *HLA-G* haplotypes by NGS from position −1983 to +3447 both in a cohort of 330 HI and in 580 patients from a French asthmatic multicenter cohort. sHLA-G was quantified using the ELISA test (MEM-G/9) in sera from 582 asthmatic patients and in 528 serum samples collected 1 year after the first sample. Associations between patients’ asthmatic features, sHLA-G expression, and genetic polymorphism were tested with logistic regressions.

*HLA-G* haplotypes displayed statistically significant differential distribution between HI and asthmatic patients. Furthermore, multivariate analyses showed significant association of *HLA-G* haplotypes with asthma inflammation markers (eosinophil count) and asthma severity (history of near-fatal asthma and exacerbation). We did not find any association between peripheral sHLA-G and genetic data in our asthmatic patient cohort; such an association described by us and others in physiological conditions may be masked in an inflammatory context as asthma. The lack of association between peripheral sHLA-G expression and asthmatic features supports the hypothesis that sHLA-G is not overexpressed as a systemic immune response to control local inflammation. These results reinforce the involvement of HLA-G in asthma and suggest that the modulation of local sHLA-G associated with asthma phenotypes and asthma inflammation markers described in Ref. (9) might be partly driven by *HLA-G* genetic polymorphism.

We also performed descriptive analyses on full length *HLA-G* sequences: phylogenetic analysis, *in silico* prediction of TF binding sites, and *in silico* prediction of splicing sites.

Phylogenetic analysis suggested a specific evolution of *HLA-G* haplotypes. *HLA-G* haplotype grouping based on their TF binding site profiles mirrored that observed on the phylogenetic tree.

UTR2-H10 was associated with asthmatic exacerbation within the previous 12 months and eosinophil count, and UTR7 (HLA-G*01:01:03:03-H16) was associated with acute asthma recurrence. These sequences, the closest within the phylogenetic tree and according to TF binding site profiles, could lead to similar expression regulation. These results support the hypothesis of an impaired immunomodulation of the UTR2-*HLA-G* molecule, previously associated with a worse evolution of cystic fibrosis (23) and pregnancy complications (37, 38) for individuals carrying UTR2-HLA-G*01:06.

Transcription factor binding site analysis supported a specific evolution of haplotype H23 that displayed an impaired PR site compared to other haplotypes. Progesterone is thought to play a major role in HLA-G expression because of concomitant requirement of both molecules for embryo implantation and pregnancy success. *In vitro* studies showed that progesterone enhanced HLA-G expression (39, 40). This unique feature of haplotype H23 may lead to an impaired activation of local HLA-G expression and a loss of local inflammation control. Haplotype H23 (Fq = 7.4%) was associated in the asthmatic patient cohort with higher eosinophil count in univariate analysis (*p* = 0.04); however, multiple correction testing did not reach statistical significance (pc = 0.22). One may speculate, however, that a larger cohort may lead to statistical significance; thus this haplotype might be a good candidate marker for inflammation, as we previously associated UTR3-HLA-G*01:04 with impaired long-term survival following lung transplantation (23). Haplotype H23 is split into H23-UTR3-G*01:04:01 and H23-UTR3-G*01:04:04, respectively, higher and lower in HI than in asthmatic patients (*p* = 0.044 and *p* = 0.012). These sequences differ at position 1827G>A in the last positions of exon 4 which, according to *in silico* predictive analysis, creates an exonic cryptic acceptor site. We previously reported alternatively spliced isoform expression of HLA-G in HBEC from asthmatic patients as compared to HI forms (10). These results support the hypothesis that the mRNA transcript of these HLA-G*01:04 alleles might be processed differently leading to varied protein expression but could also explain discrepancies of clinical studies with *HLA-G* typing at a four-digit level.

H04-UTR4-G*01:01:01:05 and H02-UTR4-G*01:01:01:05 were both higher in HI than in asthmatic patients (*p* = 0.015 and *p* = 0.009). No specific TF binding site was predicted by *in silico* analysis that could account for a putative protective effect; however, it could be due to a specific splicing process: when compared to its closest sequences (clade 4, Figure 1), HLA-G*01:01:01:05 displayed, according to *in silico* predictive analysis, cryptic sites that are disrupted in *HLA-G* *01:01:01:01, HLA-G *01:01:01:04, and *HLA-G* *01:01:01:06.

In conclusion, our results suggest that *HLA-G* phylogeny reflects *HLA-G* haplotype-specific association with different clinical conditions: clades 1, 2 and 3 gathered *HLA-G* sequences associated with immune impairments in diverse pathological conditions (Figure 1) (3, 10, 23). This study would benefit from the replication of pertinent results in additional cohorts to support the pertinence of defining *HLA-G* haplotype as a predictive genetic marker for asthma. Whereas the mechanisms with which HLA-G expression modulates asthma severity or phenotype are still poorly understood, it appears clearer that bronchial HLA-G expression is a more reliable marker than systemic and peripheral expression [(9) and our data]. However, given the discomfort of taking BAL samples and cross-reactivity in HLA-G antibody capture assays, *HLA-G* haplotype sequencing offers a reproducible qualitative marker that can be obtained from a sampling method which is easy to implement. Our results support a phylogenetic evolution of *HLA-G* sequences associated with differential qualitative and/or quantitative levels of protein expression, potentially driven by variations in TF binding sites and alternative splicing sites. Post-transcriptional mechanisms, not explored in this study, might also modify translation and shedding of sHLA-G and thus may further explain the lack of correlation observed between peripheral sHLA-G and genetic data in our asthmatic patient cohort.

This descriptive study does not address the putative role of HLA-G in impaired control of bronchial epithelium immune response and/or pro-inflammatory and pro-angiogenic responses described at the maternal fetal interface (2, 41, 42), nor did we provide any experimental evidence for HLA-G haplotype actual involvement in HLA-G expression.

## Ethics Statement

This study was carried out in accordance with the French Public Health Code (art L1221-1). All subjects gave written informed consent in accordance with the Declaration of Helsinki. Asthmatic patients were enrolled in a French asthmatic multicenter cohort [Cohorte Obstruction Bronchique et Asthme (COBRA)].

## Author Contributions

CaR, FC, CeR, FJ, PG, GM, NM, JP, CP, JC, LA-R, PC, DG, JDC: substantial contributions to the conception or design of the work; or the acquisition, analysis, or interpretation of data for the work. CaR, FC, CeR, FJ, PG, GM, NM, JP, CP, JC, LA-R, PC, DG, JDC: drafting the work or revising it critically for important intellectual content; final approval of the version to be published; agreement to be accountable for all aspects of the work in ensuring that questions related to the accuracy or integrity of any part of the work are appropriately investigated and resolved.

## Conflict of Interest Statement

The authors declare that the research was conducted in the absence of any commercial or financial relationships that could be construed as a potential conflict of interest.

## Abbreviations

HLA, human leukocyte antigen; CTL, cytotoxic T-lymphocyte; NK, natural killer; SNP, single-nucleotide polymorphism; sHLA-G, soluble HLA-G; UTR, untranslated region; URR, upstream regulatory region; HBEC, human bronchial epithelium cells; VBMD, volunteer bone marrow donors; BMI, body mass index; NGS, next-generation sequencing; ACT, asthma control test; ICU, intensive care unit.
